# Special Issue “Properties and Applications of Nanoparticles and Nanomaterials: 2nd Edition”

**DOI:** 10.3390/ijms26146763

**Published:** 2025-07-15

**Authors:** Xiaogang Li

**Affiliations:** Institute of Nuclear and New Energy Technology, Key Laboratory of Advanced Reactor Engineering and Safety of Ministry of Education, Tsinghua University, Beijing 100084, China; xiaogangli@mail.tsinghua.edu.cn

Nanomaterials have rapidly developed, and attention surrounding their use has increased in recent years. The emergence of various nanomaterials, i.e., nanoparticles, nano-grained alloys, and gradient nanostructures, is expected to make it possible for materials with super or very special properties to be applied in unusual practical contexts. There is a wide range of applications for nanomaterials in biochemistry and molecular medicine, including fuel cells or metal-ion batteries, flexible electronics, and various components related to energy. The physical and chemical properties of nanostructures are determined by their chemical composition, structure and formation processes, which is critical for their reliability and use in practical applications.

The purpose of this Special Issue is to provide a research forum to report on the structure, properties, processing, and applications of nanoparticles and nanomaterials to explore more possibilities to address intractable challenges. Topics of interest include but are not limited to, the areas mentioned above. Other relevant studies, such as the design of novel nanostructures or the modification of nanoparticles, have also been included. The contributors to this Special Issue, comprising outstanding scholars and industrial researchers, have put forward cutting-edge and in-depth scientific views to help our colleagues deepen their understanding of the problems facing nanoparticles and nanomaterials and address these intractable challenges.

Cea-Callejo et al. contributed a paper entitled “Point-of-Care Diagnostic Test for Rapid Detection of Infectious Laryngotracheitis Virus by Loop-Mediated Isothermal Amplification and Nanoprobes”, which details the development of a rapid, point-of-care test using LAMP and DNA–gold nanoparticle probes to detect the Infectious Laryngotracheitis Virus (ILTV) in chickens without DNA extraction, providing a visible colorimetric result. This cost-effective method achieves 100% sensitivity/specificity, detects 200 viral copies in 45 min, and enables early on-farm outbreak control to enhance poultry biosecurity.

Mohebbi et al. contributed a paper entitled “Tunable Optical Properties of Cu/VSe_2_ from the Visible to Terahertz Spectral Range: A First-Principles Study”, which reveals that while the monolayer’s metallic characteristic persists, Cu placement (top/corner) shifts electronic states near the Fermi level and significantly alters optical properties—enhancing Re (ω) in the visible range and introducing strong THz absorption peaks (0.2 THz for Top, 2.9 THz for Corner) in Im (ω). This was determined using DFT/DFTB calculations on Cu/VSe_2_ interfaces. The Cu layer also increased the refractive index (5.4 for Top and 4.6 for Corner vs. 2.9 for pure VSe_2_) and boosted the electrical current in Au/Cu/VSe_2_/Au and Ag/Cu/VSe_2_/Ag nanodiodes compared to Cu-free devices.

Etefa et al. contributed a paper entitled “Applications of Green Carbon Dots in Personalized Diagnostics for Precision Medicine”, which introduces the exceptional potential of Green Carbon Dots (GCDs) in precision medicine, enabling precise non-invasive diagnostics, real-time monitoring, and targeted therapy delivery via integration with genomic, proteomic, and bioinformatics platforms. The unique optical properties and biocompatibility of GCDs allow for tailored treatment strategies and enhanced patient-specific applications, significantly improving diagnostic accuracy and therapeutic outcomes. The ongoing integration of GCDs with advanced technologies promises the further revolutionization of personalized healthcare, paving the way for more effective individualized medical care.

Basu et al. contributed a paper entitled “Advancements in Lithography Techniques and Emerging Molecular Strategies for Nanostructure Fabrication”, which reports that lithography enables semiconductor scaling through advanced techniques like high-resolution EUV, maskless EBL/IBL, cost-effective NIL, and synchrotron-based XRL—each addressing specific needs in resolution, throughput, or prototyping for ICs, photonics, and MEMS/NEMS. Despite persistent challenges (defect control and material limits), innovations in alignment marks, mask fabrication, and molecular strategies ensure that lithography remains indispensable for pushing nanoscale manufacturing boundaries.

Martínez-Cisterna et al. contributed a paper entitled “Chitosan-Coated Silver Nanocomposites: Biosynthesis, Mechanical Properties, and Ag+ Release in Liquid and Biofilm Forms”, in which chitosan-coated silver nanoparticles (AgChNPs) were biosynthesized using the Galega officinalis extract and optimized (1% chitosan, pH 4), achieving a size of 207.88 nm, +42.30 mV zeta potential, and spherical morphology (5.54–61.46 nm), with FTIR confirming functionalization. AgChNP-integrated biofilms demonstrated tunable mechanical strength (3.48 MPa tensile and 24.99 mm elongation), controlled silver ionization (62.57 mg/L in liquid; 184.07 mg/kg in films), and thermal stability, highlighting their potential for controlled-release pest management in sustainable agriculture.

Broset et al. contributed a paper entitled “A Complete Approach for circRNA Therapeutics from Purification to Lyophilized Delivery Using Novel Ionizable Lipids”. Optimized purification and delivery methods were developed for circular RNA (circRNA), including high-yield Oligo(dT) affinity chromatography and novel lipid nanoparticles (LNPs) formulated with ionizable lipids (CP-LC-0867/CP-LC-0729), which significantly enhanced protein expression with sustained activity over 14 days compared to conventional SM-102 lipids. The successful lyophilization of LNP-circRNA complexes using CP-LC-0729 maintained therapeutic efficacy without cold-chain storage, demonstrating scalable strategies to overcome critical stability, purification, and delivery barriers for practical circRNA therapies.

Taha et al. contributed a paper entitled “Silver Nanoparticles as a Novel Tissue Preservative: A Comparative Study with 10% Neutral Buffered Formalin”. The authors claim that silver nanoparticles (AgNPs) outperform formalin in preserving DNA, RNA, and protein integrity across multiple murine tissues over 72 h but are less effective at maintaining tissue morphology. This supports AgNPs as a viable molecular-preserving fixative, pending improvements in structural preservation for broader histopathological use.

Kim et al. contributed a paper entitled “Cardiovascular Toxicity of Metal-Based Nanoparticles”. The authors note that metal-based nanoparticles (MNPs) pose cardiovascular risks due to systemic translocation after inhalation, ingestion, or dermal exposure, accumulating in tissues and disrupting endothelial function via oxidative stress, apoptosis, ferroptosis, and barrier impairment. Their nanoscale properties and ion release synergistically drive toxicity—increasing endothelial permeability, compromising the blood–brain barrier, and enhancing procoagulant activity—contributing to vascular/cardiac dysfunction and cardiovascular diseases. This review synthesizes recent in vitro and in vivo evidence on MNP biodistribution and cytotoxic mechanisms, providing a comprehensive assessment of their pathological impact on the cardiovascular system.

Loskutova et al. contributed a paper entitled “Quantum Dots-Based Luminescent Sensors: Review from Analytical Perspective”. This review critically analyzes luminescent quantum dot (QD)-based sensors, synthesizing data from 124 studies to reveal that chemiluminescent QDs achieve exceptional sensitivity (average LOD: 0.109 pM), outperforming fluorescent (38 nM) and phosphorescent (26 nM) variants. While AI integration (e.g., ATTBeadNet and SVM) enhances analytical performance, key challenges persist in recovery rates, biocompatibility, and stability, guiding the future development of robust QD sensors for diverse analyte detection.

Tomic et al. contributed a paper entitled “Targeting Osteosarcoma: The Dual Action of Halogenated Boroxine and Cerium Oxide Nanoparticles”, which has been approved for publication by us and will be available online soon. Addressing the limitations of decades-old osteosarcoma therapies, the authors evaluate the novel agents of halogenated boroxine (HB) and dextran-coated cerium oxide nanoparticles (SD2), both individually and in combination in an in vitro model, assessing cytotoxicity, viability, oxidative stress, cell death, and the effects on 3D spheroids. The results of this work demonstrate dose-, time-, and cell type-dependent antitumor effects for both agents, primarily via cytotoxicity and specific ROS induction, with combination treatment yielding modulated responses (potentially synergistic/selective) and significantly reduced toxicity towards non-tumor cells in 3D models, suggesting a promising selective strategy warranting further mechanistic investigation at the transcriptomic/proteomic levels.

This Special Issue highlights novel approaches and views and is committed to the effective application of nanoparticles and nanomaterials. We sincerely thank the authors who submitted manuscripts to our Special Issue as well as our readers. We hope that this Special Issue will serve as a platform to promote dialog among scientists in the field of nanoparticles and nanomaterials. In addition, in view of our continued intake of high-quality articles on this topic, we have begun a new Special Issue, “Properties and Applications of Nanoparticles and Nanomaterials: 3rd Edition”; the submission address is https://www.mdpi.com/journal/ijms/special_issues/HWH2W8ZA94 (accessed on 5 July 2025). Topics of interest include but are not limited to the structure, properties, processing, and applications of nanoparticles and nanomaterials, the design of novel nanostructures, and the modification of nanoparticles. The submission of research articles and reviews in this area of study is welcome.

